# Drilling down on the vitamin D debate: where are our priorities?

**DOI:** 10.1186/1687-9856-2015-S1-O21

**Published:** 2015-04-28

**Authors:** Leanne M Ward

**Affiliations:** 1 University of Ottawa, Ottawa, Ontario, Canada

## 

Vitamin D deficiency remains a global, public health concern despite intense focus in recent years. While nuanced issues such as the most appropriate cut-offs to define degrees of vitamin D sufficiency, insufficiency and deficiency are still unsettled, these debates occur on a backdrop of children continuing to be diagnosed around the world with the most devastating consequences of overt vitamin D deficiency - rickets and hypocalcemic seizures. The fact that few, if any, countries are exempt from new cases of vitamin D deficiency rickets underscores the potential barriers to effective prevention, including well-publicized national policies, education at multiple levels of the care system, access to vitamin D supplementation and compliance. As pediatricians, the global eradication of childhood rickets due to vitamin D deficiency is arguably our most important mandate at the moment, one that should not be overshadowed by the vitamin D adequacy cut-off debate. Only a small, critical amount of vitamin D daily is required post-natally to prevent rickets, with the optimal approach to rickets prevention targeting mother-infant dyads. Single dose or intermittent therapy is an appealing strategy to overcome compliance issues, although a full understanding of the risks related to higher dose therapy during the early years is still needed. Overall, this presentation will focus primarily on issues and strategies related to the global eradication of vitamin D deficiency rickets, with some discussion around the optimization of vitamin D status throughout the pediatric years.

